# Implications of Blending Pulse and Wheat Flours on Rheology and Quality Characteristics of Baked Goods: A Review

**DOI:** 10.3390/foods11203287

**Published:** 2022-10-20

**Authors:** Sunday J. Olakanmi, Digvir S. Jayas, Jitendra Paliwal

**Affiliations:** Department of Biosystems Engineering, 75 Chancellors Circle, University of Manitoba, Winnipeg, MB R3T 5V6, Canada

**Keywords:** food quality, composite flour, baking characteristics, quality characteristics, composite flour, protein substitution

## Abstract

Bread is one of the most widely consumed foods in all regions of the world. Wheat flour being its principal ingredient is a cereal crop low in protein. The protein content of a whole grain of wheat is about 12–15% and is deficit in some essential amino acids, for example, lysine. Conversely, the protein and fibre contents of legume crops are between 20 and 35% and 15 and 35%, respectively, depending on the type and cultivar of the legume. The importance of protein-rich diets for the growth and development of body organs and tissues as well as the overall functionality of the body is significant. Thus, in the last two decades, there has been a greater interest in the studies on the utilization of legumes in bread production and how the incorporation impacts the quality characteristics of the bread and the breadmaking process. The addition of plant-based protein flours has been shown to produce an improved quality characteristic, especially the nutritional quality aspect of bread. The objective of this review is to synthesize and critically investigate the body of research on the impact of adding legume flours on the rheological attributes of dough and the quality and baking characteristics of bread.

## 1. Introduction

Breadmaking has been described as one of the oldest practices known to mankind. It is believed to be a major part of the diet of the people of Babylon, Egypt, Greece and Rome for many decades before the present era. The first bread was produced around 10,000 years BC or over 12,000 years ago and was a result of the deliberate test with water and grain (wild wheat and wild barley) and plant roots flours [1,2]. The ability to control the production of bread and its distribution is described as a way of exerting political power for about 2000 years and its scarcity has been likened to difficult times [3]. Bread is a light porous solid material which is traditionally produced from wheat flour. Wheat (*Triticum aestivum*) is a cereal crop which constitutes about 20% of the calories consumed by humans all over the world [4]. The two most important factors contributing to the choice of bread by all as a key food commodity are: (i) the simplicity of its ingredients and method of production and (ii) the wide array of cereals that can be used to bake it. The principal ingredients for bread making are flour (mostly wheat flour), water, a leavening agent (yeast or other chemicals) and sodium chloride. These ingredients are properly mixed to form a dough. The mixing operation is carefully carried out such that the dough possesses the required mechanical properties that allow it to hold gas and a well-developed bread loaf with a uniform crumb structure is produced [3,5,6,7]. With the right ingredients and baking process, products with excellent quality and sensory characteristics are produced. Freshly prepared bread mostly has an attractive brownish and crunchy outer crust, a pleasing roasty aroma, good crumb porosity, excellent slicing properties, a moist mouthfeel and soft and elastic crumb textural characteristics [8].

People in different parts of the world consume bread in different shapes and forms with an average consumption of 70 kg per year per capita. There are thousands of recipes for bread making and this accounts for the wide array of bread types available [5]. The types of bread baked around the world include unleavened, sourdough, French, brown or whole meal, wheat germ, high protein, high fibre, multigrain, soft grain, ethnic multigrain, slimming and health high fibre, added malt grain, cereals other than wheat (including from gluten-free raw materials), crisp, meeting special dietary needs and war and famine bread [9]. This large number of bread types confirms that human beings have different recipes for bread baking and how its consumption has influenced humanity, with profound origins in religious beliefs, conflict management and well-being [5,9].

In the last few decades, there is a progressive increase in the consumption of bread in many developing countries. This trend can be attributed to changing eating habits and a constant increase in the population [10]. In many developed countries; however, there has been a significant decline in the intake of certain types of bread, specifically the white bread types. The change in the consumer behaviour can be traced to the consumer awareness of the quality of the product, consumer perception of bread, the desire for protein-rich diets and the desire for gluten-free diets. Research has shown that wheat, which is a major and the most common flour used for bread baking is considerably low in health-stimulating bioactive compounds such as vitamins, β-carotene, polyphenols, dietary fibre and flavonoids. Additionally, being a cereal crop, its protein content is relatively low (12–15%) compared to legumes (20–35%). Legumes/pulses rich in protein include chickpeas, common beans, dry beans, cowpeas, faba beans, lentils, lupin, mung beans, mesquite pods, peanuts, pigeon peas and soybeans [11,12,13,14]. Proteins, like other biological macromolecules such as carbohydrates, are important components of organisms and partake in almost every activity inside the organism’s cells. They are of great nutritional importance and partake directly in the chemical processes essential for life including catalyzing metabolic reactions, DNA replication, response to stimuli, production of hemoglobin, provision of structures to body cells and moving different molecules from one part of the body to another [12]. Other proteins perform different functions which include cell signalling, immune responses, adhesion of cells, and the cell cycle [15]. 

This trend has birthed a greater interest among researchers, policymakers and food product developers in the studies on the utilization (partial substitution) of legumes or pulses flour in bread making and how this impacts bread quality characteristics [5,16]. Therefore, the objective of this review is to synthesize knowledge from studies that explore: (i) the types of legumes/pulses that have been used for bread baking (ii) the impact of protein substitution on the rheological features of bread dough, (iii) the impact of protein substitution on the baking properties and quality attributes of bread, and (iv) explore the use of flours from other sources, e.g., cereal crops other than wheat and root and tuber crops in bread baking and other pastry products.

## 2. Flour

The English word “flour” was derived from the Old French word fleur or flour, which means “the finest”. Flours are obtained upon the elimination of coarse and unwanted substances from grain during the milling process. They are fine powdery materials produced from raw grains, roots, beans, nuts or seeds after the grinding and sifting operations. Traditionally, the milling process is accomplished with a grinding stone or steel wheel. However, in the modern era, this has been replaced with roller mills. Flours contain a high amount of carbohydrates; also called polysaccharides, with starches dominating [17,18]. 

### 2.1. Wheat Flour

**Figure 1:**
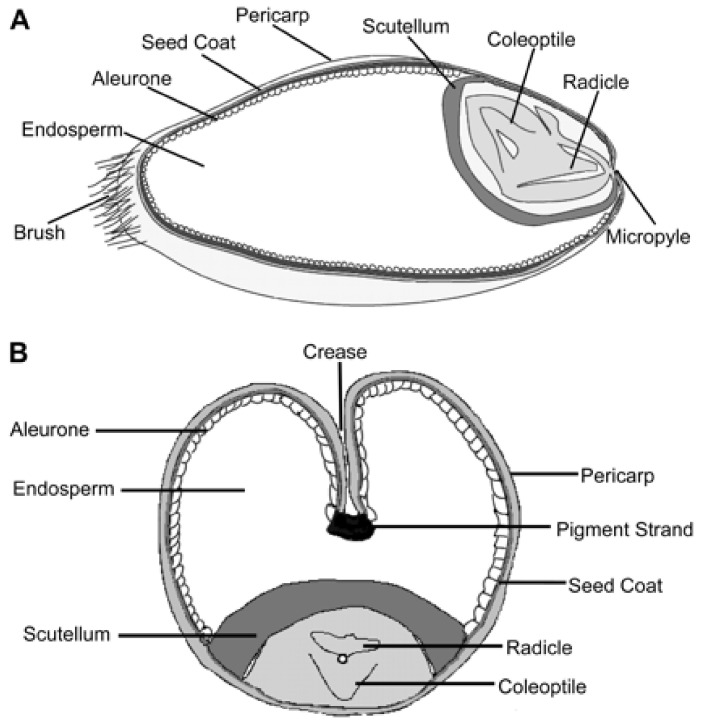
Diagram of a whole wheat grain revealing main structures in (**A**) Longitudinal and (**B**) Transverse sections, identifiable in the Magnetic Resonance Micro-Imaging images [20]; Open access.

Wheat is a cereal crop which accounts for one-fifth of the calories consumed by humans all over the world [4,19]. The wheat kernel (Figure 1) is made up of three components: (i) the endosperm, which constitutes about 80–85% of the kernel and is rich in protein and starch), and (ii) the germ (or embryo), which makes up about 2–3% of the kernel and is rich in protein, fat, vitamins and (iii) the bran, which constitutes about 13–17% of the kernel and rich in fibre. Wheat flour is a powder obtained from the grinding and sifting of wheat grains [4].

The milling process is designed to produce mainly white flour. White or refined flours are generally obtained from wheat grains containing only the endosperm (i.e., both the bran and the germ have been removed). Whole wheat flour on the other hand is made from whole wheat grains (i.e., all three components are milled together to produce the flour). Germ flours are made from both the endosperm and germ with the exclusion of the bran [21]. Hardness is the term used to describe the resistance of the endosperm to grinding. The starch granules are generally closely packed in the protein matrix and adherence between starch and protein is usually strong in hard wheat. Most hard wheat varieties are known to contain a higher amount of protein than do most soft wheat flours but protein concentration does not necessarily depend on hardness as it differs remarkably within a class and even within a given cultivar [19]. With respect to the gluten content, wheat flour is categorized as “soft” or “weak” if the gluten content is low (7–12%) and “hard” or “strong” when the gluten content is high (12–18%). Hard kinds of wheat are generally suitable for bread making due to the large amount and great quality of gluten formed when mixed with water whereas soft kinds of wheat are particularly better for cakes, pastries and cookies [21].

**Figure 2:**
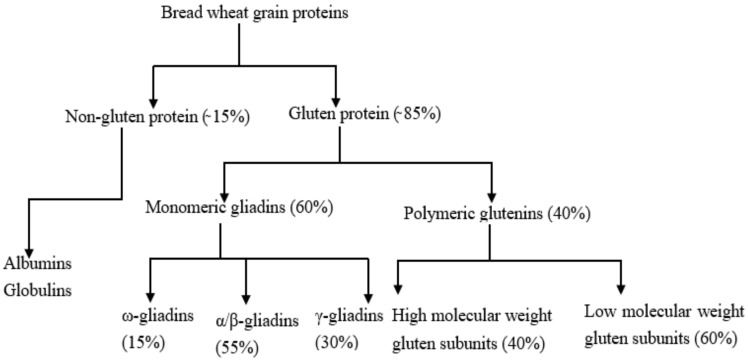
The composition and classification of wheat proteins modified from [25]; Open access.

The endosperm, which is the main component of white flour consists of two types of proteins: gluten proteins and water-soluble proteins. The gluten proteins account for about 85% of wheat protein and the remainder is the water-soluble proteins. This combination is responsible for the unique bread-making properties of wheat flour [22]. The two major proteins present in gluten are gliadin and glutenin. Based on their chemical characteristics, gliadins and glutenins have some similarities and differences. They both contain a high amount of glutamine and proline and a low amount of lysine, though the gliadins tend to have more glutamic acid, proline and amino acids with hydrophobic side chains and a slightly lower content of basic amino acids than do the glutenins [23]. Glutenins; however, have a much higher molecular weight than gliadins. Gliadin molecules are compact because of intramolecular disulphide bonding but glutenin molecules are relatively extended and highly associated because of intermolecular disulphide bonding. Gliadins and glutenins both have a high water-absorbing capacity. Both together take up nearly three times their weight of water. Gliadins, when hydrated separately, become sticky and readily extensible. Glutenins are both cohesive and elastic. However, when hydrated together, they produce gluten; which is a viscoelastic network. Albumins and globulins are water-soluble proteins of wheat endosperm. Together they constitute only 10–15% of the total protein. Their major contribution to baking quality lies in the enzymes. Some of the enzymes include amylases (β-amylases and α-amylases) and other proteolytic enzymes, e.g., lipoxidase, lipases, and phytase [24]. The composition and classification of the wheat proteins are shown in Figure 2.

Wheat flour has been used for different purposes. The presence of gluten, the dough-forming constituent of wheat flour, accounts for its applications in the production of leavened products [22]. With the addition of water to the wheat flour, a dough with unique rheological characteristics with the ability to retain gas bubbles is formed. This also accounts for its viscoelastic and thermosetting properties. Additionally, the baking characteristics of wheat flour are a function of the gluten protein [26]. Because of these unique properties, wheat flour has been used for making bread, noodles, pasta, cookies and cakes and is more popular than any other cereal grain for use in baked goods.

Despite the great properties of wheat flour and its suitability for bread making, research has shown that it contains a lower amount of health-promoting bioactive molecules like vitamins, β-carotene, polyphenols, dietary fibre and flavonoids [27]. Additionally, some adverse reactions are associated with the consumption of gluten-rich products (gluten protein), especially in people with celiac disease [26]. For these people, the most effective method of treatment is strict abstinence from foods containing gluten proteins [28]. Efforts have been made globally to explore alternative flours that could partially or completely replace wheat flour in the production of wheat flour-based products [16]. 

### 2.2. Composite Flours

Composite flour is described as a mix of flours, starches and other ingredients in which wheat flour is completely or partly replaced with flours from other sources in bakery and pastry products. This can be a mixture of two or three kinds of flour from either plant or animal sources with or without the addition of wheat flour [10]. These flours have economic potential for both developing and developed countries and make a substantial nutritional contribution [19]. Common plant-based flours that have been used for the production of wheat flour-based products in different parts of the world include different legumes/pulses [29,30,31,32,33], different cereal crops [34,35,36,37] and some roots and tuber crops [38,39,40,41].

#### 2.2.1. Use of Legumes and Pulses in Bread Baking and Pastry Foods

Legumes are defined as the edible seeds of the family Leguminosae or Fabaceae, the second-largest family of seed plants containing 600 genera and about 13,000 species. The word ‘pulses’ (also called grain-legumes) is derived from the Latin word, *puls*, meaning ‘a pottage made of meal’. One of the most important characteristics of legumes is the presence of high amounts of proteins. These proteins have high lysine content, an essential amino acid needed by the body. Proteins in legumes are deficient in Sulphur-containing amino acids, making them an excellent addition to other commonly used cereal proteins (e.g., wheat) which are low in lysine, but rich in Sulphur amino acid content [10,30,42]. The amino acids profile of common pulses is shown in Table 1. Pulses are also known as a great source of bioactive compounds which have tremendous advantages to human health in addition to substances that have exceptional functional properties to foods and food commodities [30,43]. 

Nutritionally, pulses contain a relatively high amount of protein (20–35%) and fibre (15–20%) as compared with cereal grains and lower amounts of fats (below 10%) [44]. The nutritional profile of common pulses is summarized in Table 2. Globally, soybeans are the most commonly consumed legume crop, followed by peanuts, dry beans, dry peas, chickpeas, cowpea, faba beans, lentil, pigeon pea, navy beans, pinto beans, miscellaneous beans, lupin, and Bambara beans [14,45]. 

Different legumes have been blended with wheat and other cereal flour to produce different kinds of bakery and pastry products. Notably include: 

Faba beans (*Vicia faba*) (also known as broad beans or fava beans) which has been used to produce bread [46], spaghetti [32], corn-based pasta-like products [47] and pasta [48].

Cowpea (*Vigna unguiculata)* (also called black-eyed peas, southern peas, or crowder peas) is used in the production of bread [49] and macaroni [50].

Soybean (*Glycine max*) has high lysine content and this makes it an ideal crop to improve the essential amino acids profile when blended with cereal crops. It has been used to produce bread [51], noodles [52] and pasta [33].

Bambara groundnut (*Vigna subterranean*) has been used in the production of bread [53] and pasta [54].

Mesquite flour is defined as a sweet and aromatic product produced from milling the whole ripe fruit of the mesquite tree (*Prosopis spp*.). Bigne et al. (2018) used mesquite flour at 150–350 g/kg mixed with wheat flour at 850–650 g/kg to produce composite sweet bread [55].

Lentil (*Lens culinaris*) was used by Perri et al. (2021) to produce bread. They used a blend of wheat flour and sourdough made from whole and sprouted lentil flour. They reported that processes like fermentation and germination are potential approaches to enhance the use of legumes in novel foods [57]. Other products that have been produced with a blend of lentil flour include cake [58] and cookies [31].

Yellow pea flour (*Pisum sativum*) has been used to produce biscuits [59].

Chickpea (*Cicer arietinum*) has been used to produce bread [42,60], cake [61], spaghetti [62], pasta [63] and noodles [64].

Common bean (*Phaseolus vulgaris*) was used to produce spaghetti pasta with a blend of Mexican common bean flour and semolina [65]. 

Pigeon pea (*Cajanus cajan*) is also one of the nutritionally important legumes of the tropical and subtropical regions of the world. Its application for pastry products was explored by Rafiq et al. (2017) who produced pasta from semolina flour substituted with legume flour and brown rice flour using a hot extrusion process (twin screw extruder) [66]. 

Fluted pumpkin (*Telfairia occidentalis*) seed flour was used to produce bread with a blend of wheat flour and fluted pumpkin seed flour [67]. A summary of the utilization of legumes in baked products is given in Table 3.

##### Rheological Characteristics of Dough and Impacts of Protein Substitution on Dough Characteristics

Rheology is defined as the study of the deformation and flow of materials. It involves the study of how a given material reacts to applied stress or strain. Different instruments used to measure the rheological properties of dough include a penetrometer, consistometer, amylograph, farinograph, mixograph, extensigraph, retetexturom, maturograph, oven-rise recorder and alveograph. Results of these empirical tests depend on the instrument type, the geometry and size of the sample being tested and the conditions under which the test was carried out. Bread doughs are known to exhibit viscoelastic and shear thinning properties, combining the properties of Hookean solids and non-Newtonian viscous liquids. Dough exhibits a non-linear rheological characteristic, but when subjected to minimal strain produces a linear behaviour. The extent of low strain that a dough exhibits linearity is a function of the dough type and the methods of mixing and testing [68].

The storage modulus, loss modulus and loss tangent describing a material’s rheological properties are defined as follows:

(1) $$\;G^{\prime }=\frac{\tau_{0}\;Cos\;\theta }{{ϒ}_{0}}$$

(2) $$\;G^{\prime \prime }=\frac{\tau_{0}\;Sin\;\theta }{{ϒ}_{0}}$$

(3) $$tan\;\delta =\frac{G^{\prime \prime }}{G^{\prime }}$$

where: G′ depicts the storage modulus, G″ is the loss modulus and tan δ is the loss tangent.

The storage modulus defines the elastic properties of a sample and the loss modulus defines the viscous properties of a sample [68,69].

Gluten, the principal protein present in wheat dough exhibits viscoelastic property where the gliadin portion represents viscous property and the glutenin components represent elastic behavior resulting from the variation in their molecular sizes. Increasing the protein in dough produces higher consistency and improving intermolecular cross-linkage lead to higher G′ and a reduced loss tangent in the dough. The relationships (which include physical and chemical attractions) among the protein molecules significantly contribute to the rheological characteristics of dough [68]. The rheological property of wheat gluten is the basis of its functional properties and makes it different from all other commercially available plant proteins. These properties allow it to be used to produce bread, cakes, biscuits and noodles [70]. Rheological characteristics of dough also have a profound impact on the quality of the product as well as process efficiency. These properties can be correlated to the mechanical properties and the specific volume of bakery products. The mechanical properties (e.g., compression, tension, shear) of bread crumbs are important for periodic quality assurance in the baking industry and to assess the impact of changes in dough ingredients and baking conditions. The compression test, which is a measure of bread firmness is used to evaluate the mechanical properties of bread crumbs and it relates to the subjective methods of touch or mouthfeel. This property has been demonstrated to have a positive correlation with the sensory attributes of the baked product. Tensile test on the other hand is hardly used to measure the mechanical properties of bread and other spongy foods because it is challenging to grip the food sample, inability to meet compliance at the grips and inability to obtain the size, shape and stiffness stipulated for the test in those food materials [3]. It has been established that the texture and density of baked goods for instance bread and cakes are influenced by variations in their rheology and vapour content during baking [2].

The dough rheology and quality of bread depend largely on the starch-protein complex, and most importantly, the presence of gluten. The addition of non-wheat flour for baked goods can have negative effects on the gluten network, leading to weakened bread dough and degradation in bread quality characteristics [71]. The major problem with non-wheat grain flours is the result of their weak dough viscoelastic and gas-holding properties resulting from the lack of gluten [72]. 

Water absorption capacity is an essential property that indicates a flour’s ability to absorb water and produce dough of excellent consistency. The impact of legumes/pulses addition on the water absorption capacity of dough has been extensively studied. The addition of chickpea, soybean, common bean, fava bean and lentil flours to wheat flour has been reported to increase the water absorption capacity of dough compared to wheat dough [42,73,74,75]. This can be attributed to the water absorption capacity of the gluten, protein particle entrapment inside the gluten network structure and the likely relationship between the gluten and some of the legume proteins probably present on the outer surface of the hydrated particles [42,73].

The addition of the enzyme transglutaminase has been reported to reinforce the protein network and induced a significant increase in the water absorption capacity of rice flour, soy flour and pea protein isolate blends, producing a synergetic effect and a reduction in the storage (G′) and viscous (G′′) moduli. The main function of the enzyme transglutaminase is to covalently crosslink proteins through the association between an ε-amino group on protein-bound lysine residues and a γ-carboxamide group on protein-bound glutamine residues. [29]. Different processing methods like heat treatments and germination have been shown to positively influence the functional properties of both legumes and cereal seeds [76,77]. The toasting of yellow peas flour resulted in improved dough water absorption capacity and enhanced stability of the dough, giving rise to bread with increased specific volume and loaf density comparable with 100% wheat flour control [78]. 

The inclusion of wheat-lupin protein isolates has been reported to enhance the development time of dough, its strength and resistance to deformation and extensibility. This was a result of the lupin particle entrapped inside the gluten network structure, and a likely correlation between the gluten and some of the lupin proteins present in the outer part of the moistened particles [79]. The inclusion of chickpea flour with wheat flour in the production of bread increased in development time of dough, while there was a reduction in the extensibility and the deformation resistance of dough. The topmost part of the wheat dough as well as the blend with 10% chickpea flour were categorized as “normal”, nevertheless, the blend containing 20 and 30% resulted in a “sticky” dough surface. The chickpea addition improved the dough development time and stability and the extensograph properties of the dough [42]. Adding chickpea, lentil and bean flour to wheat flour leads to an improvement in the development time of the dough and a reduction in dough stability [43,75]. Olapade and Oluwole (2013) reported a significant increase (*p* < 0.05) in the functional characteristics, excluding the bulk density and swelling capacity of composite flour produced from wheat flour partially substituted with 10% acha flour and 0–15% cowpea flour compared with that produced from 100% wheat flour [49]. 

According to Kahraman et al. (2018), raw, roasted and dehulled chickpea flours increased the viscous and elastic moduli of rice-based dough, leading to a good structuring of the dough. A reduced retrogradation tendency of the slurry comprising chickpea flours was also confirmed by the viscoamylographic test. This is a promising outcome for baking food applications [80]. Baiano et al. (2011) reported that the substitution of semolina with toasted and partly defatted soy flour resulted in dough weakening and an increase in the tenacity-extensibility ratio. The authors reported that the struggle for water in soy proteins with starch and gluten positively influenced the spaghetti with respect to cooking and overcooking resistance, compensating for the deleterious impacts resulting from the partial decrease in the gluten network and the resulting dough weakening [81].

Other processing techniques such as the usage of food hydrocolloids, enzymes, and sourdough fermentation have been demonstrated to improve the functionality of the dough and bread textural quality of pan-type bread produced from non–wheat flours [72]. The inclusion of hydrocolloids with different chemical structures in the production of noodle-based goods has been proposed to enhance the textural properties of noodles in addition to compensating for the decreased quality of the final products as a result of the reduced gluten content [82]. The addition of a low level of *Artemisia sphaerocephala* Krasch gum (ASKG) (0.03–0.5%) caused a significant improvement in the viscoelastic characteristics of the composite dough system, followed by a reduced trend at a higher level of gum inclusion (0.8%). Addition of the gum at 0.03–0.5% increased dough G′/G″ values. The addition of 0.3% of the gum resulted in a relatively denser and more arranged network structure of the dough while 0.5% and 0.8% of the gum resulted in the disruption of the strong network with visible signs of starch deformation [83]. Therefore, hydrocolloids are very useful for improving the quality of dough made from mixtures of wheat flour and non-wheat flour.

In addition, Wang et al. (2018) reported that the addition of microbial dextran (synthesized in situ from *W. confusa*) to faba beans sourdough containing dextran improved the viscoelastic properties of the dough, improved the specific volume, and decreased crumb hardness of the bread produced as compared with the unblended sample [84]. Marco & Rosell (2008) reported a decrease in the storage (G′) and viscous (G″) moduli when different structuring agents: hydroxypro- pylmethylcellulose and a processing aid; transglutaminase were used to modify the rheological properties of soybean-enriched rice doughs [29]. Huang et al. (2019) also reported that rheological assessment showed that the inclusion of tempeh flour (TF) increased G′ and G″ moduli of dough. They concluded that the addition of tempeh flour increased the volume and viscoelastic characteristics of the dough. It also led to a reduction in moisture migration rate and water loss in bread crumbs [85].

In addition, it has been shown that there is a need for the inclusion of different additives to the blends to obtain the desired gluten-like structure when non-wheat flour is used for bread making. The classes of additives used in breadmaking include oxidants/reductants (e.g., Azodicarbonamide, Ascorbic acid), emulsifiers (e.g., Mono- and diglycerides, Diacetyl tartaric acid esters of mono- and diglycerides, Lactylates: calcium stearoyl-lactylate and sodium stearoyl-lactylate) and hydrocolloids (e.g., Xanthan gum, Guar gum) [86] For example, β-conglycinin concentrate, which is obtained after fractionation of soybean proteins was assessed in a lean system in which other additives were not used in the production of bread. The bread produced had greater 2D area, height, softness and cohesiveness in comparison with vital gluten bread [86]. The addition of 10% β-conglycinin concentrate produced from defatted soybean flour to rice flour on bread quality characteristics was studied by Espinosa-Ramírez et al. (2018). They reported that the inclusion of 10% β-conglycinin concentrate in rice flour formulation for bread making led to bread comparable to vital gluten bread. From the micrograph analysis, they reported that the inclusion of β-conglycinin created a net-like structure comparable to the one created by gluten, affirming the capability of β-conglycinin of behaving as a structuring agent and an improver of protein quality in gluten-free bread preparations [26].

##### Impacts of Protein Substitution on Baking Characteristics of Bread

Baking creates sequences of chemical, physical, and biochemical reactions, producing different changes in the end product characteristics such as volume enlargement, moisture evaporation, formation of porous structure, protein denaturation, starch gelatinization, formation of crust and browning reaction, protein cross-linking, melting of fat and crystals and their incorporation into the outer layers of air cells, gas cells rupture and fragmentation of cell walls [6,8,87]. The combined processes of gas production, moisture evaporation and change in the rheological properties of the dough results in gas retention loss, transforming the dough’s foam structure into an open sponge structure of bread with interconnected cells. There is a continuous modification of the bread flour because of these activities until the structure of the final product is achieved. Factors affecting this stage include temperature, humidity and the duration of baking [8]. Other changes that occur in bread during baking are changes in physical dimensions (i.e., volume, height, width and length of the bread loaf), texture modification and moisture changes, colour modification and flavour generation [6]. It has been reported that excess of some ingredients, for example reducing sugars similar to amino acids improved the nonenzymatic Maillard browning reactions, leading to the formulation of crust and darkening. 

In addition, functional characteristics of food proteins such as the ability to foam and water retention can be improved with heat treatment in the presence of sugars; complex carbohydrates by the process known as the Maillard reaction [88,89]. The denatured whey protein has been demonstrated to enhance the baking performance and texture of wheat bread dough [90]. Table 4 summarizes the impacts of protein substitution on the baking characteristics of bread.

##### Impacts of Protein Substitution on Bread Quality Characteristics

Quality is defined as the combination of distinguishing characteristics and properties of a food commodity that can determine its degree of acceptance by a user or consumer [91]. Bread quality depends greatly on consumers’ perception, which is a function of different factors including the social, demographic and environment of an individual. Bread quality can be grouped into different subcategories: (i) instrumental attributes–features that can be objectively measured, (ii) sensory attributes–features that relate to consumers’ perceptions or judgement and (iii) nutritional attributes–those related to health-promoting effects and functionality of the bread [6,25]. Sensory at-tributes are usually correlated and compared to objective physical measurements [6]. 

Examples of different attributes of bread that can be objectively measured and have been quantified to determine the quality of bread include volume (using rapeseed dis-placement), specific weight, specific volume, moisture content, water activity, crust and crumb colour, crust crispiness, crumb hardness, cell distribution within the loaf slice using image analysis, and volatile composition. The attributes related to sensory sensations of bread include visual appearance, taste, odour and tactile and oral texture [25]. The impacts of protein substitution on the quality characteristics of bread have been extensively studied. Partial substitution of wheat flour with legume-based proteins had effects on the nutritional/proximate, physical and sensory quality characteristics of bread. Table 5 summarizes the impacts of protein substitution on bread quality characteristics.

#### 2.2.2. Use of Other Cereals for Bread Making and Other Pastry Products

Cereals are grass crops grown for the edible components of their grains. They are regarded as staple foods and an essential source of micronutrients such as vitamins, minerals and macronutrients such as proteins, carbohydrates, fibre, crude fats, and essential fatty acids, which perform important functions in the health of humans. The extensively consumed grains are wheat, maize, rice, and barley while minimally consumed grains are rye, sorghum, quinoa, oat, and millet [102,103]. Cereal flour is mostly utilized to produce baked products such as bread, cakes, pastries, and cookies also including additional food commodities such as spaghettis, noodles, confectionery products, and infant foods [104].

Among the commonly studied cereal crops used to produce bread and other pastry products is sorghum. The addition of sorghum flour has been reported to result in a gradual decrease in composite bread [105,106]. This is due to low down amounts of the dough’s gluten network, resulting in a reduction in the ability of the dough to rise; as a result of the weaker cell wall structure leading to bread having low specific volume when contrasted with wheat flour bread [107]. Bread produced entirely from sorghum needed an alternative bread-production improvement and the addition of hydrocolloids [108]. Jafari et al. (2018) produced dough and bread using sorghum-wheat composite flour and added xanthan gum at 0.5 and 1%. They reported that extruded sorghum-wheat dough had the highest heating rates (10.75 °C/min) and non-extruded sorghum-wheat dough comprising 0.5% xanthan gum produced the lowest (7.33 °C/min) heating rates [109]. 

Quinoa is also a widely studied cereal crop in the production of pastry products. Bilgicli (2013) reported that the addition of pseudo-cereals, e.g., quinoa at 25% of a recipe in gluten-free noodles can enhance nutrient content, for instance, proteins and minerals (calcium, magnesium, zinc, and iron) [110]. Giménez et al. (2016) reported that the substitution of maize flour with quinoa flour in the production of pasta-like products showed an additive effect, remarkably enhancing the dietary fibre contents, unsaturated fatty acids, iron, and zinc [47]. Schoenlechner et al. (2010) reported that the inclusion of quinoa flour improved the cooking losses of gluten-free pasta [111]. Tiga et al. (2021) reported that the addition of quinoa flour improved the water absorption, hardness, and redness (a*) values and reduced the cohesiveness and luminosity (L*) values of instant noodles produced [40]. Alvarez-Jubete et al. (2009) reported a significant increase in the bread volumes of buckwheat and quinoa bread in contrast with the control (rice flour and potato starch). Moreover, the pseudo-cereal-containing breads were characterized by a significantly softer crumb texture that was due to the presence of natural emulsifiers in the pseudo-cereal flours [35]. Cárdenas-Hernández et al. (2016) reported that pasta with amaranth ingredients had reduced cooking time, improved cooking loss percentage, reduced luminosity values and increased nutrient content when contrasted with semolina control pasta [112]. 

Another cereal crop which has been used in the production of pastry products is millet. Chaitra et al. (2020) produced Belgian waffles with wheat flour substituted with finger millet and pearl millet flours and reported that the control sample (100% wheat flour) had a harder texture with a shear force of 35.86 N compared with the blended samples with a shear force ranging from 19.68 N to 27.02 N. The result of the sensory analysis proved that the samples containing millet flour were more accepted, up to 50% [113]. Ibidapo et al. (2020) reported a significant increase in the nutritional properties (dietary fibre, calcium, phosphorus and sodium) of bread produced from wheat flour (65.18%) mixed with malted millet flour (19.43%) and okra flour (15.39%) [114]. Torbica et al. (2019) also reported that wheat flour substituted at 60% with barley flour led to an increase in insoluble fibre, soluble fibre, total phenolic compounds and antioxidant activity by 700%, 200%, 41.5 and 45%, respectively. They concluded that the inclusion of sesame seeds can increase the acceptability of barley-enriched bread by consumers in addition to the inherent health benefits [115]. Al-Attabi et al. (2017) reported a corresponding decrease in protein and gluten contents when barley was added to wheat flour to produce bread while the ash content and enzyme activity increased [116]. 

Lin et al. (2012) produced bread from wheat flour partially substituted with micro-fluidized corn bran at 18, 20, and 22% of flour. They reported that for the three kinds of bran substitution when the moisture content was improved from its standard values of 38.3, 38.6, 38.8% to 40.8, 41.9, and 44.0%, correspondingly, the loaves obtained showed comparable microstructure, specific loaf volume, and textural characteristics as the unblended bread [117]. Jeong et al. (2017) produced dough with rice flour substituted with rice flour-zein in a hydrated viscoelastic state at 5 and 10% by weight to account for the functionality of wheat gluten in the gluten-free sheeted dough. There was an increase in the mixing stability and development time of the rice dough with progressive amounts of zein substitution [118]. Storck et al. (2013) reported that protein-fortified, gluten-free baked foods with enhanced crumb texture and improved specific volume could be achieved with the addition of transglutaminase (1.35 U/g of protein), albumin (0.67/100 g) and casein (0.67/100 g) [119]. 

Renoldi et al. (2021) reported that pasta produced from psyllium seed husk was firmer and sticker than 100% durum wheat semolina. The cooking loss was reported to have increased with increasing levels of psyllium seed husk substitution above 25 g/kg with values below the technologically acceptable limit of 80 g/kg. Replacement of semolina with 50–100 g/kg psyllium seed husk was potent in reducing the predictive glycemic response of supplemented pasta in comparison with the unfortified sample and this was attributed to the formation of fibre aggregates limiting starch swelling after the scanning electron microscopy and dough rheology [120]. 

#### 2.2.3. Use of Root and Tuber Crops in Bread Making and Other Pastry Products

Roots and tuber crops are an essential component of the human diet as they are the main source of energy in the form of carbohydrates for the body. There are enormous kinds of roots and tuber crops produced globally. Nevertheless, their extensive utilization in the food industry is limited to only a small number of common types such as potato, cassava, sweet potato, yams and taro [121,122]. Orange-fleshed sweet potato is a biofortified variety of sweet potato that is high in β-carotene, a precursor of vitamin A and other health-promoting bioactive compounds like flavonoids, dietary fibre, vitamins and polyphenols [27,123,124]. Chikpah et al. (2021) reported that partial replacement of wheat flour at 29.4 or 28.0% of peeled or unpeeled, orange-fleshed sweet potato flour, respectively and baking temperature of 180 °C for 15 min produced the best quality dough and bread quality features [27]. Cui & Zhu (2022) reported the addition of purple-fleshed potato can result in Chinese steamed bread with improved nutritional quality and phenolic profiles [41].

Idowu et al. (1996) reported a reduction in oven springs and specific volumes when cocoyam flour was blended with wheat flour to produce bread [38]. Chisenga et al. (2020) reported that wheat can be substituted with cassava flour up to 10% in bread making without negatively impacting the overall bread quality [125]. Jensen et al. (2015) reported that depending on the type of cassava flour, wheat flour can be replaced with cassava flour up to 30% with the addition of psyllium husk (7%) without any significant differences from 100% wheat flour bread [39]. Shittu et al. (2009) reported that the inclusion of xanthan gum to a cassava-wheat flour blend had substantial effects on the dough firmness and extensibility and sensory satisfactoriness of bread and the storage stability of bread [126]. 

Nyembwe et al. (2018) also reported that the defatted marama flour mixed with cassava flour at a ratio of 33:67 can yield a dough of similar strength, but with reduced stability compared with wheat flour dough [127]. Nindjin et al. (2011) reported that white wheat flour replacement with yam starch up to 30% or cassava starch up to 20% led to kinetics expansions of resulting doughs comparable with the unblended sample. The results of the sensory analysis suggested that 30% yam starch replacement and 20% cassava starch resulted in bread that met consumer satisfaction on all the quality characteristics of the unblended sample [128]. 

## 3. Conclusions

Pulses are eco-friendly, nutrient-dense and widely cultivated crops all over the world. Due to their high nutrient profiles, they are useful tools to compact undernutrition in low-income countries and malnutrition in developed countries of the world. Wheat flour is the traditional flour used in bread making and other pastry products. This review has summarized reports on the recent applications of legumes/pulses in the production of baked and pastry products, impacts on the rheological qualities of dough and effects on the baking and quality attributes of bread. Wheat flour is a cereal crop and is a deficit in some nutrients highly required for body growth and functionality. Therefore, from this review, it has been shown that the addition of pulse flour to wheat flour in bread making is a viable means to enhance the nutritional profile of the products. However, from the reviewed literature, most of the studies reported focused on just the particle size of the legume flours and a limited study was reported on the impact of particle size on the quality and baking characteristics of the bread produced from the composite flours. Therefore, more research should be conducted on the potential effects of different flour particle sizes on the quality attributes of bread and the overall acceptability by consumers.

## Figures and Tables

**Table 1:** Amino acids (mg) composition of some pulses (expressed per 100 g edible portion on a dry matter basis).

Common Name	Alanine	Arginine	Aspartic Acid	Cysteine	Glutamic acid	Glycine	Histidine	Isoleucine	Leucine	Lysine	Methionine	Phenylalanine	Proline	Serine	Threonine	Tryptophan	Tyrosine	Valine
Adzuki bean	1190	1320	2430	190	3190	779	540	815	1720	1540	216	1080	900	1000	694	197	609	1050
Bambara Groundnut	856	1270	2130	144	3250	552	394	870	1510	1220	399	946	831	1140	834	115	449	836
Broad bean	1000	2380	2720	303	4240	1050	652	1010	1810	1570	166	1070	1010	1180	870	217	802	112
Chickpea, desi	922	2170	2370	646	3840	798	600	893	1600	1420	220	1190	1090	1190	773	217	626	908
Chickpea, kabuli	817	1660	2110	267	3900	802	637	817	1470	1220	272	1200	969	1020	831	213	609	824
Common bean	971	1270	2520	104	3380	898	590	760	1480	1300	185	1000	921	1220	881	234	638	961
Cowpea	1050	1580	2540	114	3960	928	766	1040	1760	1510	405	1260	1040	1100	891	253	649	1190
Kidney bean	996	1420	2900	242	3160	1160	602	1110	1970	1670	271	1310	654	1180	1040	328	905	1400
Lentil	1270	1830	3190	229	4710	1020	557	971	1850	1710	200	1110	1210	1100	773	239	694	1220
Lima bean	1070	1280	2700	231	2960	883	639	1100	1800	1400	264	1200	950	1390	903	248	740	1260
Lupin	1120	3160	3380	610	7930	1360	884	1350	2460	1650	215	1330	1350	1640	1160	281	1130	1300
Moth bean	1200	1310	2490	104	3570	965	698	1070	1750	1470	325	1110	1040	1080	924	231	650	1170
Mung bean	1020	1390	2490	161	3750	1440	559	605	1480	1160	224	1010	980	1390	772	208	560	937
Mungo bean	1060	1500	2960	180	4220	952	674	1020	1890	1550	313	1400	1030	1260	757	241	736	1160
Pea	1040	2030	2690	263	4020	1030	568	931	1670	1640	224	1110	967	1130	884	206	762	1100
Pigeon pea	1040	1350	2140	234	3760	766	685	780	1520	1360	253	1740	890	921	759	173	668	950
Pinto bean	911	1200	2360	98	3180	844	554	714	1390	1220	173	944	865	1140	827	220	599	903
Wheat flour (control)	543	696	802	309	4839	579	379	556	1112	416	251	772	1594	759	442	130	352	687

Source: Food and Agriculture Organization of the United Nations, (2016, Javaloyes et al., Pulses: Nutritious Seeds for a Sustainable Future). Reproduced with permission [56].

**Table 2:** Energy and macro-components of mature, whole, dried, raw pulses (expressed per 100 g edible portion on a dry matter basis).

Common Name	Energy (kcal)	Water (g)	Protein (g)	Carbohydrates (g)	Fibre (g)	Fat (g)	Ash (g)
Bambara Groundnuts	325	9	18.4	33.7	28.9	6.4	3.4
Broad bean	309	10.9	25.5	38.3	20.8	1.4	3.3
Adzuki bean	318	11	20.5	51.3	13.1	0.6	3.7
Chickpea, desi	332	10	21.2	40	21.2	5	2.7
Chickpea, kabuli	359	8.5	20.8	48.9	13.1	6.1	2.8
Common bean	305	10.4	20.9	40.7	22.6	1.5	3.8
Cowpea	324	10.6	22.5	46.9	14.6	1.9	3.5
Kidney bean	307	10.9	22.8	39.4	21.7	1.6	3.6
Lentil	324	9.7	24.4	44.8	17	1.5	2.7
Lima bean	316	9.2	20.9	45	19.1	1.5	4.2
Lupin	309	9.4	34.1	10.8	35.3	6.5	3.8
Moth bean	326	9.6	23.9	45.9	14.9	1.9	3.8
Mung bean	325	9.7	20.9	49.6	15.4	1.3	3.1
Mungo bean	316	9.8	23.9	42.2	19.5	1.4	3.4
Pea	310	11.3	23.4	38.4	22.2	2.1	2.7
Pigeon pea	306	11.4	20.6	41	21.4	1.8	3.8
Pinto bean	301	12.4	19.6	43.8	18	1.3	4.9
Wheat flour (control)	341	12.2	12.1	69.4	1.4	1.7	2.7

Source: Food and Agriculture Organization of the United Nations, (2016, Javaloyes et al., Pulses: Nutritious Seeds for a Sustainable Future). Reproduced with permission [56].

**Table 3:** Summary of the utilization of legumes and pulses in bread baking and pastry foods.

Composite Flours	Products Produced	Mixing Proportion	Acceptable Mixing Ratio	References
Faba beans & Wheat flours	Bread	25, 30, 35 and 40%	40%	[46]
Faba beans & Wheat flours	Spaghetti	10, 20 and 30%	30%	[32]
Faba beans & Wheat flours	Pasta	10, 30 and 50%	30%	[49]
Faba beans, corn & quinoa flours	Corn-based pasta-like product	30%	30%	[47]
Wheat, acha & Cowpea flours	Bread	0–15%	10%	[49]
Wheat & Cowpea flours	Macaroni	20% (*w*/*w*)	20%	[50]
Wheat, full-fat lupin, soya & triticale flours	Bread	5 and 10% *w*/*w*	5 or 10%	[51]
Wheat, Sorghum & Soy flours	Noodles	13.20%	13.20%	[52]
Rice & Soyabean flours	Soy-rice pasta	10–30%	15%	[33]
Wheat, acha & Bambara nut sourdough flours	Bread	5, 10 and 15%	10%	[53]
Wheat & Bambara nut flours	Pasta	20%	20%	[54]
Wheat & Mesquite flours	Bread	150–350 g/kg	250 g/kg	[55]
Wheat & Lentil flours	Bread	30% *w*/*w* sourdough	30% *w*/*w*	[57]
Wheat, navy bean, pinto bean, green lentil & yellow pea flours	Cookies	25, 50, 75 & 100 g/100 g	75 g/100 g	[31]
Wheat &Yellow pea flours	Biscuit	10–50%	30%	[59]
Wheat & Chickpea flours	Bread	10 to 30%	10%	[42]
Wheat & fractionated Chickpea	Bread	20–30% *w*/*w*	-	[60]
Wheat & Mexican Common bean	Spaghetti Pasta	15% and 30%	-	[65]
Wheat, Pigeon pea & brown rice flours	Pasta	10–30%	-	[66]
Wheat & Fluted pumpkin flours	Bread	10, 20, 40 & 50%	20%	[67]

**Table 4:** Impacts of protein substitution on baking characteristics of bread.

Legumes	Mixing Proportion	Effects on Baking Characteristics	References
Soybean (full-fat & defatted) & barley flours added to wheat flour	5, 10, 15 and 20% substitution levels	-decrease in loaf volume and specific loaf volume, -change in the colour of the crumb from creamish white to dull brown, -progressive hardening of crumb texture with an increase in substitution level, -increase in the loaf weight and denser bread texture with an increase in substitution.	[92]
Acha and cowpea flours added to wheat flour	10% acha flour and 0–15% cowpea flour	-decrease in the average loaf height with an increase in substitution level, -decrease in loaf volume & specific loaf volume with the protein addition, -increase in loaf weight with an increase in substitution level.	[49]
Lentil and bean flour added to wheat flour	10, 20 & 30%	-reduction in volume, specific volume, and cambering with the addition of legume flours, -legumes addition above 10% negatively impacted the shape, crust colour, crumb elasticity, and hardness of the final products.	[75]
Full fat lupin, soya &triticale flours added to medium strength wheat flour	5 and 10% *w*/*w*	-decrease in loaf volume with the addition of lupin & soy flours as a result of the dilution of the gluten structure by the incorporated proteins, -decrease in dough height and bread yield (g/100g of flour) with the addition of medium strength -darkening of the crust colour; the crumb colour became more yellowish & crumb texture showed evidence of thickened cells.	[51]
Faba bean flour added to 2 varieties of hard red spring wheat (Neepawa & Glenlea)	about 20% protein	-decrease in loaf volume in both wheat varieties.	[93]
Acha and Bambara nut sourdough flours added to wheat flour	0, 5:5, 10:10 and 15:15	-specific volume, colour and texture of composite bread were not significantly different from the control.	[53]
Mesquite flour added to wheat flour	150–350 g/kg added to 850–650 g/kg wheat flour	-the addition resulted in lower loaf heights (up to 41%) and firmer crumb -crumb microstructure showed smaller & more irregular alveoli with thicker walls with the addition of mesquite flour.	[55]
Chickpea flour added to wheat flour	10, 20 & 30%	-the colour of crust and crumb increasingly got darker as the level of chickpea flour substitution increased, -decrease in baking loss, loaf height, loaf volume & specific loaf volume as the level of substitution increased.	[42]
Chickpea flour, pea isolate, carob germ flour or soya flours each added with corn starch, xanthan gum	94 g chickpea, 24.4 g pea isolate 60.2 g soya flour 47.2 g carob germ flour	-carob germ flour bread gave the lowest specific volume values (2.51 cm^3^/g) however chickpea bread gave the highest (3.26 cm^3^/g), -chickpea bread had the softest crumb, -carob germ flour bread had a more compact microstructure compared with soya & chickpea formulations, -no significant differences were observed in bake loss & water activity values of the four bread samples.	[94]
Faba bean flour added with wheat flour	25, 30, 35 & 40%	-significant (*p* < 0.05) decrease was recorded for the lightness and whiteness index with the increase in substitution level.	[46]
Faba beans, carob & gluten added to wheat flour	5.72, 2.86 & 1.43% respectively	-decrease in bake loss (%), cell area (%), slice brightness, lightness of crust and crumb with the addition of legume flour, -increase in loaf hardness & number of cells in the composite flour bread.	[95]
Faba bean protein isolate added to wheat flour	5–8%	-pale crust colour and a slightly negative effect on loaf volume & crumb grain in the composite bread.	[96]
Sprouted lentil & wheat flour	30% *w*/*w*	-increase in specific volume and reduction in crumb hardness and staling rate in the composite bread compared with the control	[57]
Fermented chickpea flour & wheat flour	20%–30% *w*/*w*	-decrease in loaf-specific volume and the crumb structure became denser with an increase in chickpea flour replacement, -with sourdough addition, the crust showed less browning, compared to others.	[60]
Fluted pumpkin seed & wheat flours	10, 20, 40 & 50%	-increase in loaf volume & loaf firmness with an increase in the substitution level.	[67]

**Table 5:** Impacts of protein substitution on bread quality characteristics.

Legumes	Mixing Proportion	Effects on Nutritional Quality Features	Effects on Sensory Quality Features	References
Faba beans	25, 30, 35 & 40%	-ash, proteins, minerals, total phenolic compounds, condensed tannins, total flavonoids contents & antiradical activity increased with fava bean flour addition	-composite flour, up to 40% substitution level produced acceptable quality characteristics, -composite bread was most preferred in terms of the aroma as it imparted a feeling of satiation.	[46]
Faba beans, added to wheat flour	5.72, 2.86 & 1.43%	-low contents of anti-nutritional compounds, -improved amino acid profile & protein efficiency ratio, -increased nitrogen utilization (by 69%), -high antioxidant potentials linked to high phenolics	-scored higher in terms of crumb moisture, -good consumer acceptance in terms of colour, odour, taste, -composite bread scored significantly higher in elasticity & lower in adhesiveness.	[95]
Acha & cowpea added to wheat flour	10% acha flour & 0–15% cowpea flour	-increased in proximate composition with an increase in substitution i.e., crude protein, crude fibre, fat & ash	-loaves from supplemented composite flours with up to 10% cowpea flour were acceptable by the panellists.	[49]
Soybean (full-fat& defatted) & barley flours added to wheat flour	5, 10, 15 and 20%	-increased protein & glutelin contents	-10% soy flour or 15% barley flour gave acceptable organoleptic properties.	[92]
Soy, plantain & wheat flours	5,10 &15%	-increased proximate properties, especially protein, fibre with increased soy flour levels	-5% soy flour addition produced acceptable organoleptic properties like the control.	[97]
Soy flour & wheat flour	10, 20 & 30%	-improved nutrient contents with the addition of soy flour.	-the bread gave acceptable sensory properties in blends below 30% soy flour addition.	[98]
Mesquite flour & wheat flour	5, 10 & 15%	-improved nutritional quality with an increase in fibre contents & unsaturated fatty acids. -retarded staling of the composite bread	-----	[99]
Sprouted lentil & wheat flours	30% *w*/*w*	-improved nutritional quality, especially total & soluble fibers.	-improved sensory (i.e., synthesis of key-aroma compounds) quality of the final products	[57]
Fractionated Chickpea flour & wheat flour	20–30% *w*/*w*	-38.5% (on a dry basis) increase in the protein content with 30% *w*/*w* replacement. -levels of raffinose, stachyose, and verbascose in the sourdough bread was reduced by 75.4, 97.6 & 90.0% compared to control.	-	[60]
Acha & Bambara nut sourdough & wheat flours	5:5, 10:10 & 15:15	-significant increase in proximate properties (crude protein, dietary fibre, protein digestibility, mineral, amino acid profile, -increase in antioxidant contents in the composite bread -decrease in the amount of antinutrients (phytate, tannin)	-substitution of up to 10% sourdough flour in bread significantly improved taste, flavour & acceptability scores compared to wheat bread.	[53]
Fluted pumpkin seed & wheat flours	10, 20, 40 & 50%	-increase in proximate composition (protein, crude fibre, fat, ash, carbohydrate and moisture contents)	-crust colour, crumb texture, taste, flavour, appearance & general acceptability showed that 10% and 20% levels of substitution were both acceptable.	[67]
Lupin flour treated with ultrasound & traditional method added to wheat flour	0, 10, 15 and 20%	-Ultrasound resulted in higher volume & specific volume and lower weight, firmness, hardness and chewiness when compared with traditional method, -Lupin flour addition resulted in bread with decreased volume and specific volume when compared with 100% wheat flour, -Increased nutritional profile (protein etc.) in the composite bread.		[100]
Heat–modified cowpea protein added to wheat flour	2, 4 & 6%	-Improved nutritional quality (protein and fibre contents).	-Bread has acceptable quality up to 4%.	[89]
Mesquite flour added to wheat Flour	0 to 70 g per 100 g wheat flour	-Increased fibre content in the enriched samples	-An appealing colour & flavour in the enriched samples.	[101]
Yellow pea flour & wheat	30%	-Increased protein content (8.4% in 100% wheat flour to 10.1–10.8% in the enriched samples.		[78]

## Data Availability

Not applicable.
